# Findings of a Nationwide Mixed-Methods Assessment of Cancer Care and Prevention Needs in Botswana

**DOI:** 10.1200/GO.22.45000

**Published:** 2022-05-05

**Authors:** Kirthana Sharma, Refeletswe Lebelonyane, Tlotlo Ralefela, Peter Vuylsteke, Reena Antony, Morongwa Legwaila, Tapologo Leselwa, Tiny Masupe, Tendani Gaolathe, Richard Marlink

**Affiliations:** ^1^ Rutgers Global Health Institute, New Brunswick, NJ; ^2^ Botswana Rutgers Partnership for Health, Gaborone, Botswana; ^3^ Princess Marina Hospital, Botswana Ministry of Health and Wellness, Gaborone, Botswana; ^4^ University of Botswana, Gaborone, Botswana

## Abstract

High-level investment in cancer prevention and control in low- and middle-income countries is urgently needed to address the predicted surge in cancer incidence, yet few assessments of health systems have systematically identified gaps in infrastructure, training, and patient care in sub-Saharan Africa. In Botswana, we evaluated the current state of cancer care and prevention, to understand the strengths, weaknesses, and needs regarding the provision of comprehensive cancer services.

**METHODS:**

The study evaluated four regional hospitals designated as cancer sites by the Botswana Ministry of Health and Wellness to decentralize cancer services. A multi-site, cross-sectional evaluation using qualitative and quantitative methods was conducted. Focus Group Discussions with cancer patients, cancer survivors, caregivers, the general population, and healthcare workers were analyzed for emergent themes. Quantitative surveys assessed knowledge, attitudes and practices of health workers and hospital management staff, and cancer service gaps at health facilities.

**RESULTS:**

Knowledge gaps included low awareness of cancer signs and symptoms among the general population, poor knowledge of early detection and treatment among health workers, and caregivers lacked skills to support cancer patients. Cancer screening services, other than cervical screening, were limited in all sites. Diagnosis and treatment barriers included lack of specialized personnel, equipment, timely pathology services, and drug stockouts. There were low levels of confidence in cancer management, including chemotherapy, without support from oncologists. Providers reported low patient screening uptake due lack of access and patients reported fear of diagnosis. Health facilities did not routinely notify cancers to the national registry. Radiotherapy was limited to one private hospital in the capital.

**CONCLUSION:**

Decentralization of cancer services to regions will require substantial capacity building at district hospitals to strengthen fragmented screening, early diagnosis and treatment services. Health provider training needs and infrastructure gaps were substantial, with low public awareness of cancer signs, symptoms and causes.

